# Long-term effects of subthalamic nucleus deep brain stimulation on speech in Parkinson’s disease

**DOI:** 10.1038/s41598-023-38555-2

**Published:** 2023-07-15

**Authors:** Annalisa Gessani, Francesco Cavallieri, Valentina Fioravanti, Isabella Campanini, Andrea Merlo, Giulia Di Rauso, Benedetta Damiano, Sara Scaltriti, Elisa Bardi, Maria Giulia Corni, Francesca Antonelli, Francesca Cavalleri, Maria Angela Molinari, Sara Contardi, Elisa Menozzi, Alessandro Fraternali, Annibale Versari, Giuseppe Biagini, Valérie Fraix, Serge Pinto, Elena Moro, Carla Budriesi, Franco Valzania

**Affiliations:** 1 Neurology Unit, Department of Neuroscience, S. Agostino Estense Hospital, Azienda Ospedaliero-Universitaria di Modena, Modena, Italy; 2 Neurology Unit, Neuromotor & Rehabilitation Department, Azienda USL-IRCCS di Reggio Emilia, Viale Risorgimento 80, 42123 Reggio Emilia, Italy; 3 Clinical and Experimental Medicine PhD Program, University of Modena and Reggio Emilia, Modena, Italy; 4 LAM – Motion Analysis Laboratory, Neuromotor and Rehabilitation Department, San Sebastiano Hospital, Azienda USL-IRCCS di Reggio Emilia, Correggio (Reggio Emilia), Italy; 5 Division of Neuroradiology, Department of Neuroscience, Nuovo Ospedale Civile S. Agostino Estense, Modena, Italy; 6 Department of Neurology and Stroke Center, IRCCS Istituto Delle Scienze Neurologiche di Bologna, Maggiore Hospital, Bologna, Italy; 7 Department of Clinical and Movement Neurosciences, UCL Queen Square Institute of Neurology, London, UK; 8 Nuclear Medicine Unit, Azienda Unità Sanitaria Locale-IRCCS di Reggio Emilia, Reggio Emilia, Italy; 9 Department of Biomedical, Metabolic and Neural Sciences, University of Modena and Reggio Emilia, Modena, Italy; 10 Grenoble Alpes University, Division of Neurology, Centre Hospitalier Universitaire de Grenoble, Grenoble Institute of Neuroscience, Grenoble, France; 11 Aix-Marseille Univ, CRNS, LPL, Aix-en-Provence, France

**Keywords:** Movement disorders, Parkinson's disease

## Abstract

Bilateral subthalamic nucleus deep brain stimulation (STN-DBS) is an effective treatment in advanced Parkinson’s Disease (PD). However, the effects of STN-DBS on speech are still debated, particularly in the long-term follow-up. The objective of this study was to evaluate the long-term effects of bilateral STN-DBS on speech in a cohort of advanced PD patients treated with bilateral STN-DBS. Each patient was assessed before surgery through a neurological evaluation and a perceptual-acoustic analysis of speech and re-assessed in the long-term in different stimulation and drug conditions. The primary outcome was the percentage change of speech intelligibility obtained by comparing the postoperative on-stimulation/off-medication condition with the preoperative off-medication condition. Twenty-five PD patients treated with bilateral STN-DBS with a 5-year follow-up were included. In the long-term, speech intelligibility stayed at the same level as preoperative values when compared with preoperative values. STN-DBS induced a significant acute improvement of speech intelligibility (p < 0.005) in the postoperative assessment when compared to the on-stimulation/off-medication and off-stimulation/off-medication conditions. These results highlight that STN-DBS may handle speech intelligibility even in the long-term.

## Introduction

Bilateral subthalamic nucleus deep brain stimulation (STN-DBS) represents a short and long-term effective treatment in advanced Parkinson’s Disease (PD)^1,2^. However, the long-term effects of bilateral STN-DBS on axial features^3,4^ and different speech variables are still debated. After surgery, PD patients may develop heterogeneous profiles of dysarthria related to the possible spreading of current to cerebellothalamic, cortico-bulbar, cortico-spinal and pallido-fugal levels^5,6^. Moreover, it has been previously reported that speech intelligibility may worsen 1 year after surgery when compared with those under a control group under optimal medical treatment^7^. The majority of the studies regarding the effects of STN-DBS on speech focused on short-term follow-up^7–11^ while few studies have assessed the long-term effects^2,12–14^. In particular, a previous study reported a worsening of speech intelligibility at five and eight years after surgery in the off-medication condition^12^, while another acoustic study reported variation of the long-term averaged spectrum (LTAS) descriptors for reading and monologue in different stimulation conditions in the long-term after surgery^14^. The objective of this study was to evaluate the long-term (five years) effects of bilateral STN-DBS on speech in advanced PD patients using a standardized perceptual-acoustic analysis of speech. In the following paragraphs we will report the results of the study, followed by the discussion of these findings before finally describing the methodology of the study.

## Results

### Patient population

From 2012 to 2017, 40 PD patients underwent STN-DBS. Of these, fifteen subjects were excluded from the study because of missing data (eight patients), lack of consent to participate (four patients), and non-native Italian speakers (three patients). The remaining 25 PD patients with a median follow-up of five years after surgery (range 3–7 years) were included (males: 18; disease duration at surgery: 10.44 [± 4.62] years; age at surgery: 58.40 [± 5.73] years; age at PD onset 47.76 [± 5.63] years). Nineteen patients were included in the PIGD subtype, five in the indeterminate, and one in the TD subtype. Genetic assessment revealed heterozygous mutation in the GBA gene in three patients (12%). Preoperative brain-MRI revealed the presence of white matter hyperintensities of vascular origin in four patients (16%). The mean preoperative levodopa responsiveness was 62.24% (± 16.38%). A detailed description of stimulation parameters and settings is reported in Table 1, while the changes of the different speech and clinical variables in the different conditions tested are shown in Table 2. Concerning the perceptual assessment of dysarthria severity, a significant reduction of the score was found in all the three postoperative conditions tested when compared with the preoperative ones. On the contrary, no significant differences were found by comparing the different postoperative conditions with each other.

**Table 1:** Stimulation parameters at postoperative evaluation.

Stimulation parameters and settings	Total *n* = 25 N. (%), mean, [± SD]; median {range}
Frequency setting	
High frequency	18 (72.00%)
Low frequency	7 (28.00%)
Left STN	
Single monopolar stimulation	20 (80.00%)
Bipolar stimulation	1 (4.00%)
Double monopolar stimulation	4 (16.00%)
Contact 0 active as cathode	1 (4.00%)
Contact 1 active as cathode	14 (56.00%)
Contact 2 active as cathode	13 (52.00%)
Contact 3 active as cathode	2 (8.00%)
Voltage (V)	2.704 [± 0.731]; 2.800 {0.652–3.900}
Frequency (Hz)	133.600 [± 31.73]; 130.000 {70.000–180.000}
Pulse width (us)	64.800 [± 11.225]; 60.000 {60.000–90.000}
Power of stimulation	68.230 [± 31.042]; 69.680 {4.240–135.871}
Right STN	
Single monopolar stimulation	23 (92.00%)
Bipolar stimulation	0 (0.00%)
Double monopolar stimulation	2 (8.00%)
Contact 0 active as cathode	2 (8.00%)
Contact 1 active as cathode	15 (60.00%)
Contact 2 active as cathode	10 (40.00%)
Contact 3 active as cathode	0 (0.00%)
Voltage (V)	2.521 [± 0.775]; 2.700 {0.746–4.100}
Frequency (Hz)	126.320 [± 38.370]; 130.000 {60.000–180.000}
Pulse width (us)	68.800 [± 17.635]; 60.000 {60.000–130.000}
Power of stimulation	57.289 [± 31.157]; 55.608 {5.281 -121.571}

**Table 2:** Changes of speech and clinical variables over time.

Variable	No. (%); mean [± SD]; median {range}				
	Preoperative assessment		Postoperative assessment		
	Off-medication	On-medication	On-stimulation/off-medication	Off-stimulation/off-medication	On-stimulation/on-medication
Speech variables					
Speech intelligibility (%)	93.04 [± 8.17]; 94.00 {64.00–100.00}	92.04 [± 8.37]; 94.00 {62.00–100.00}	89.52 [± 15.83]; 96.00 {48.00–100.00}	84.34 [± 18.00]; 94.00 {48.00–100.00}* ^ç^	84.64 [± 18.40]; 92.00 {28.00–100.00}
Mean intensity of spontaneous speech (dB)	66.76 [± 5.96]; 67.00 **{**59.00–86.00}	66.92 [± 7.28]; 68.00 {49.00–76.00}	65.32 [± 6.87]; 65.00 **{**52.00–81.00}	63.04 [± 4.76]; 64.00 **{**56.00–70.00}	65.28 [± 6.02]; 67.00 **{**54.00–74.00}
F0 SD of spontaneous speech (Hz)	35.15 [± 20.09]; 31.12 **{**1.28–89.00}	36.81 [± 18.71]; 32.17 **{**0.80–76.00}	35.13 [± 16.12]; 31.57 {14.98–80.24}	30.03 [± 16.90]; 29.49{7.84–92.56}	32.80 [± 12.74]; 31.42{13.87–63.47}
Maximum phonation time (MPT) (seconds)	14.87 [± 5.38]; 14.00 **{**4.00–26.00}	16.48 [± 5.37]; 17.00 {7.00–26.00}	15.29 [± 7.00]; 13.00 **{**6.20–32.00}	12.30 [± 5.11]; 12.00 **{**5.00–27.00}*^£^	13.74 [± 5.47]; 13.00 **{**7.00–31.00}
Mean intensity of sustained phonation (dB)	73.04 [± 6.89]; 72.00 **{**60.00–89.00}	72.24 [± 7.06]; 72.00 **{**59.00–87.00}	68.80 [± 9.16]; 70.00 **{**52.00–88.00}	67.32 [± 7.20]; 68.00 **{**55.00–85.00}^ç^	69.20 [± 8.90]; 71.00 **{**46.00–88.00}
Count rate (sill/sec)	4.92 [± 1.31]; 4.75 {1.90–7.75}	4.82 [± 1.47]; 4.75 {1.50–7.75}	4.28 [± 1.43]; 4.25 {2.22–7.29}	4.16 [± 1.16]; 4.25 {1.46–6.38}	4.86 [± 1.66]; 4.64 {2.22–9.44}
Perceptual severity of dysarthria (1–4)	3.92 [± 0.27]; 4.00 {3.00–4.00}	3.96 [± 0.20]; 4.00 {3.00–4.00}	3.40 [± 0.70]; 3.00 {1.00–4.00}^ç,£^	3.40 [± 0.71]; 4.00 {2.00–4.00}^ç,£^	3.28 [± 0.67]; 3.00 {2.00–4.00}^ç,£^
Clinical variables					
UPDRS part I	2.04 [± 1.95]; 2.00 {0.00–8.00}		3.00 [± 2.02]; 3.00 {0.00–8.00}		
UPDRS part II	19.92 [± 5.67]; 20.00 {9.00–33.00}	7.08 [± 4.53]; 7.00 {1.00–17.00}	19.28 [± 5.37]; 21.00 {6.00–29.00}		13.52 [± 5.73]; 14.00 {4.00–23.00}
UPDRS part-III	36.64 [± 9.27]; 34.00 {25.00–62.00}	14.64 [± 7.38]; 13.00 {3.00–31.00}	29.28 [± 12.41]; 26.00 {13.00–58.00}	46.20 [± 12.81]; 47.00 {25.00–73.00}	15.80 [± 9.07]; 12.00 {5.00–38.00}
UPDRS part IV	7.13 [± 2.40]; 7.00 {4.00–12.00}		4.40 [± 2.06]; 4.00 {1.00–9.00}		
Hoehn and Yahr	2.82 [± 0.61]; 2.50 {2.00–4.00}	1.98 [± 0.42]; 0.42 {1.00–2.50}	2.78 [± 0.71]; 2.50 {2.00- 5.00}	3.64 [± 1.06]; 4.00 {2.00- 5.00}	2.40 [± 0.50]; 2.50 {2.00- 4.00}
UPDRS akinesia subscore	12.70 [± 3.18]; 12.00 {7.00–18.00}	4.48 [± 3.24]; 4.00 {0.00–12.00}	11.12 [± 5.45]; 11.00{2.00–23.00}	17.40 [± 5.93]; 18.00 {4.00–28.00}	6.44 [± 4.83]; 5.00 {0.00–17.00}
UPDRS tremor subscore	4.65 [± 4.11]; 3.00 {0.00–14.00}	1.26 [± 2.20]; 0.00 {0.00–9.00}	2.96 [± 2.35]; 3.00 {0.00–7.00}	4.68 [± 2.86]; 4.00 {0.00–10.00}	0.64 [± 0.99]; 0.00 {0.00–4.00}
UPDRS PIGD subscore	8.13[± 3.52]; 8.00 {3.00–16.00}	2.61[± 1.78]; 2.00 {0.00–7.00}	7.92 [± 3.06]; 8.00 {1.00–13.00}	9.40 [± 3.60]; 9.00 {1.00–15.00}	5.04 [± 3.44]; 5.00 {0.00–12.00}
UPDRS item 18	1.347 [± 0.57]; 1.00 {0.00–2.00}	0.608 [± 0.65]; 1.00 {0.00–2.00}	1.52 [± 0.71]; 1.00 {1.00–3.00}	1.68 [± 0.74]; 2.00 {1.00–3.00}	1.44 [± 0.65]; 1.00 {0.00–3.00}
LEDD (mg)	925.10 [± 439.522]; 1045.00 {200.00–1898.00}		817.36 [± 358.499]; 807.00 {118.00–1500.00}		
Phonemic fluency	34.47 [± 7.26]; 34.22 {18.29–46.90}		27.66 [± 10.57]; 27.85 {9.75–57.18}		
Spatial perception localization of numbers	8.55 [± 1.13]; 9.00 {7.00–10.00}		7.81 [± 8.00]; 8.00 {3.00–10.00}		
1947 colored Raven’s progressive matrices	28.78 [± 4.34]; 30.24 {21.00–35.61}		24.93 [± 5.79]; 24.59 {16.84–40.52}		
Stroop test “time”	20.07 [± 11.45]; 17.70 {8.00–47.00}		27.51 [± 19.60]; 25.12 {6.50–91.50}		
Stroop test “errors”	0.51 [± 0.65]; 0.37 {0.00–2.25}		2.70 [± 4.77]; 0.12 {0.00–15.75}		
Trail making test part B	101.77 [± 55.76]; 85.50 {33.00–274.00}		168.64 [± 131.24]; 120.00 {27.00–531.00}		

Speech variables: Friedman Test followed by Wilcoxon signed rank test post-hoc.

*LEDD* L-dopa equivalent daily dose, *MRI* magnetic resonance imaging, *PD* Parkinson disease, *PIGD* dominant postural instability and gait disorder, *SD* standard deviation, *UPDRS* Unified Parkinson’s Disease Rating Scale.

*p-value < 0.005 with respect to the on-stimulation/off-medication condition.

^ç^p-value < 0.005 with respect to the off-medication condition.

^£^p-value < 0.005 with respect to the on-medication condition.

### Primary outcomes

**Figure 1:**
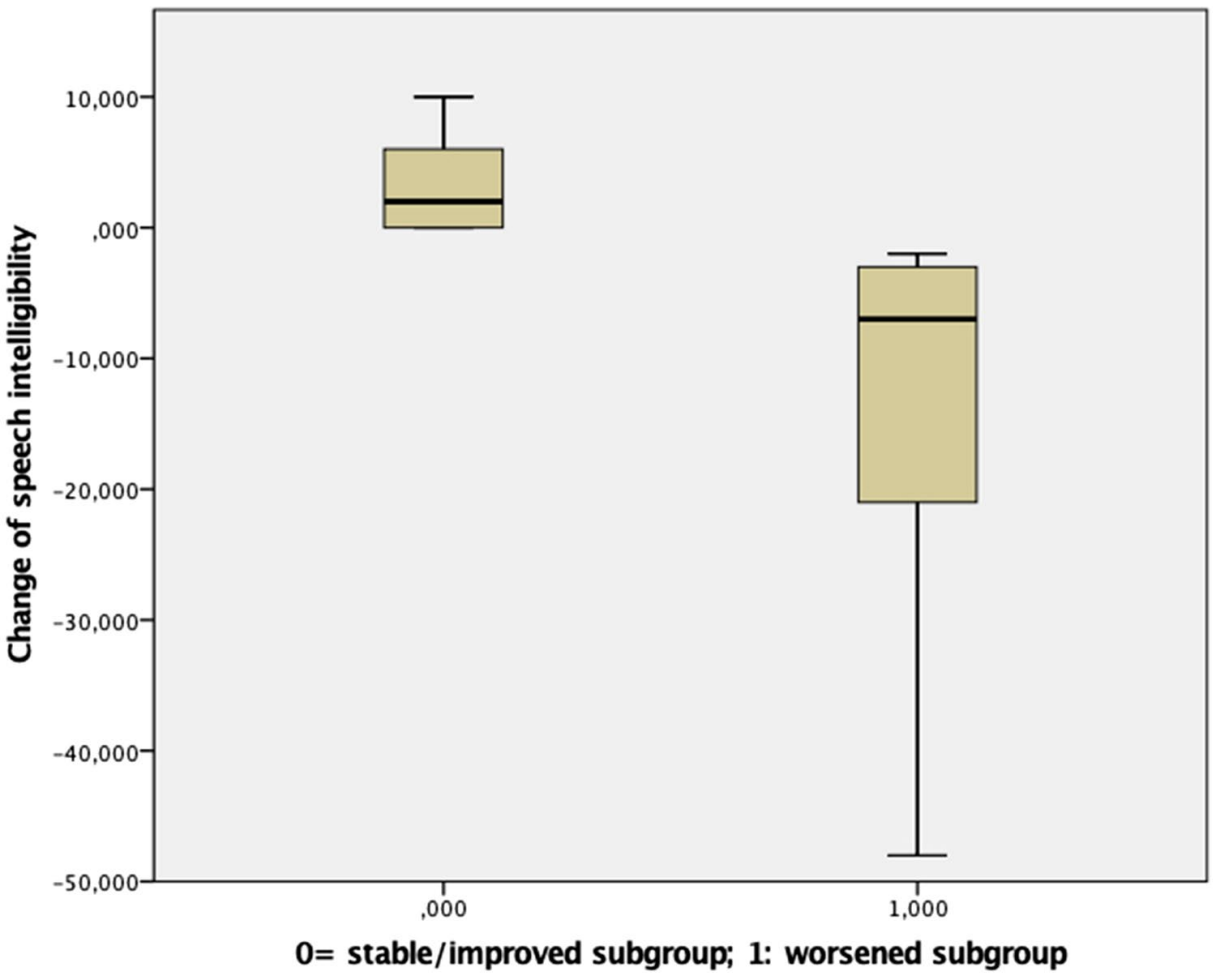
Changes of speech intelligibility in the stable and worsened groups.

In the long-term and at the group level, speech intelligibility did not significantly worsen with respect to preoperative values when comparing the postoperative on-stimulation/off-medication condition with the preoperative off-medication condition (z =  − 0.371, p = 0.710). The shutdown of the stimulation led to an acute significant worsening of speech intelligibility in the postoperative assessment (z =  − 3.500, p < 0.001) when comparing the on-stimulation/off-medication and off-stimulation/off-medication conditions. Furthermore, the assessment of disease progression effects on speech intelligibility obtained when comparing the postoperative off-stimulation/off-medication condition with the preoperative off-medication condition showed a significant worsening with respect to preoperative values (z =  − 2.92, p < 0.005). Analyzing the long-term postoperative changes of speech intelligibility at the individual level for each patient, sixteen patients were classified as “stable” while the remaining nine patients integrated a “worsened” subgroup (Fig. 1). Compared to a “stable” subgroup, “worsened” patients showed a greater preoperative and postoperative disease motor severity quantified by: (i) higher scores in the UPDRS part III total score, H&Y staging, and akinesia subscore in the off-medication condition; (ii) lower loudness of spontaneous speech and lower speech intelligibility in all three postoperative conditions; (iii) lower intensity of prolonged phonation in on-stimulation/off-medication and off-stimulation/off-medication conditions (Table 3). Furthermore, they showed a significantly worse performance in the preoperative Stroop test (errors) and postoperative Trail Making test B. In addition, all the GBA1-PD patients were included in the “worsened” subgroup with a trend toward significance (p = 0.06) at the chi-square independence test. Disease duration, age at surgery, follow-up duration and stimulation parameters were not significantly different between the two subgroups.

**Table 3:** Differences in clinical and speech variables between “worsened” and “stable” subgroups.

Variable	No. (%); Mean [± SD]; median {range}		
	“Worsened” (n = 9)	“Stable/improved” (n = 16)	P value
Preoperative variable			
Sex (male/female)	6/3 (66.7/33.3)	11/5 (68.8/31.3)	0.910
Age at PD onset (years)	49.44 [± 5.54]; 48.00 {41.00–57.00}	46.81[± 5.62]; 46.50 {38.00–55.00}	0.201
Age at surgery (years)	59.55 [± 4.15]; 60.00 {54.00–65.00}	57.75 [± 6.48]; 58.50 {46.00–71.00}	0.378
Disease duration at surgery (years)	9.66 [± 4.00]; 8.00 {5.00–16.00}	10.87 [± 5.00]; 8.50 {6.00–25.00}	0.587
Levodopa responsiveness (%)	65.14 [± 20.39]; 66.00 {34.00–93.00}	60.60 [± 14.13]; 60.71 {40.00–90.00}	0.552
LEDD (mg)	987.33 [± 377.74]; 1104.00 {350.00–1405.00}	890.09 [± 478.90]; 1023.00 {200.00–1898.00}	0.419
UPDRS part-III off-medication	43.22 [± 10.41]; 45.00 {31.00–62.00}	32.94 [± 6.24]; 32.50 {25.00–49.00}	0.016
Hoehn and Yahr off-medication	3.17 [± 0.66]; 3.00 {2.50–4.00}	2.63 [± 0.50]; 2.50 {2.00–4.00}	0.038
UPDRS akinesia subscore off-medication	15.50 [± 2.07]; 15.00 {12.00–18.00}	11.20 [± 2.62]; 11.00 {7.00–16.00}	0.002
UPDRS akinesia subscore on-medication	6.37 [± 3.89]; 5.00 {1.00–12.00}	3.47 [± 2.42]; 3.00 {0.00–8.00}	0.054
UPDRS PIGD subscore off-medication	10.00 [± 3.70]; 10.00 {5.00–16.00}	7.13 [± 3.09]; 7.00 {3.00–15.00}	0.060
Genetic analysis			
Negative	6 (66.70%)	16 (100.00%)	0.060
Positive (GBA heterozygous mutations)	3 (33.30%)	0 (0%)	
Stroop test “errors”	0.81 [± 0.65]; 0.75 {0.00–2.00}	0.33 [± 0.60]; 0.00 {0.00–2.25}	0.035
Speech intelligibility (%) off-medication	89.11 [± 11.05]; 94.00 {64.00–100.00}	95.25 [± 5.20]; 97.00 {82.00–100.00}	0.102
Speech intelligibility (%) on-medication	87.55 [± 11.34]; 90.00 {62.00–100.00}	94.56 [± 4.95]; 95.00 {84.00–100.00}	0.075
Mean intensity of spontaneous speech (dB) off-medication	68.11 [± 7.80]; 68.00 {59.00–86.00}	66.00 [± 4.76]; 65.50 {59.00–73.00}	0.776
Mean intensity of spontaneous speech (dB) on-medication	64.66 [± 9.35]; 68.00 {49.00–75.00}	68.18 [± 5.78]; 68.50 {55.00–76.00}	0.495
F0 SD of spontaneous speech (Hz) off-medication	38.41 [± 16.43]; 30.44 {22.68–61.63}	33.51 [± 22.00]; 31.11 {1.28–89.00}	0.462
F0 SD of spontaneous speech (Hz) on-medication	39.72 [± 12.97]; 40.87 {22.10–61.47}	35.35 [± 21.24]; 29.00 {0.80–76.00}	0.358
Maximum phonation time (MPT) (s) off-medication	15.11 [± 5.13]; 14.00 {8.00–26.00}	14.73 [± 5.67]; 13.85 {4.00–26.00}	0.842
Maximum phonation time (MPT) (s) on-medication	14.55 [± 4.44]; 15.00 {7.00–21.00}	17.56 [± 5.66]; 18.50 {7.00–26.00}	0.165
Mean intensity of sustained phonation (dB) off-medication	72.44 [± 7.40]; 73.00 {60.00–86.00}	73.37 [± 6.82]; 71.50 {63.00–89.00}	0.977
Mean intensity of sustained phonation (dB) on-medication	71.33 [± 6.12]; 74.00 {62.00–78.00}	72.75 [± 7.68]; 72.00 {59.00–87.00}	0.609
Count rate (sill/s) off-medication	4.61 [± 0.94]; 4.75 {3.00–5.67}	5.08 [± 1.47]; 4.97 {1.90–7.75}	0.380
Count rate (sill/s) on-medication	4.73 [± 1.23]; 4.64 {2.50–6.33}	4.86 [± 1.62]; 4.82 {1.50–7.75}	0.777
Perceptual severity of dysarthria (1–4) off-medication	3.88 [± 0.33]; 4.00 {3.00–4.00}	3.93 [± 0.25]; 4.00 {3.00–4.00}	0.673
Perceptual severity of dysarthria (1–4) on-medication	3.88 [± 0.33]; 4.00 {3.00–4.00}	4.00 [± 0.00]; 4.00 {4.00–4.00}	0.182
Postoperative variable			
UPDRS part-II off-medication	21.67 [± 6.32]; 23.00 {6.00–29.00}	17.94 [± 4.42]; 18.50 {9.00–24.00}	0.023
UPDRS part-III on-stimulation/off-medication	37.67 [± 15.76]; 42.00 {14.00–58.00}	24.56 [± 6.95]; 23.00 {13.00–36.00}	0.054
UPDRS part-III on-stimulation/on-medication	22.00 [± 11.13]; 22.00 {5.00–38.00}	12.31 [± 5.42]; 11.00 {5.00–24.00}	0.017
UPDRS akinesia subscore, on-stimulation/off-medication	15.22 [± 6.46]; 17.00 {3.00–23.00}	8.81 [± 3.08]; 9.00 {2.00–14.00}	0.011
UPDRS akinesia subscore, on-stimulation/on-medication	9.78 [± 5.63]; 10.00 {0.00–17.00}	4.56 [± 3.18]; 4.00 {1.00–10.00}	0.024
UPDRS akinesia subscore, off-stimulation/off-medication	20.00 [± 7.42]; 20.00 {4.00–28.00}	15.94 [± 4.54]; 17.00 {8.00–26.00}	0.065
Mean intensity of spontaneous speech (dB), on-stimulation/off-medication	59.78 [± 5.09]; 59.00 {52.00–69.00}	68.44 [± 5.74]; 68.00 {57.00–81.00}	0.002
Mean intensity of spontaneous speech (dB), off-stimulation/off-medication	59.89 [± 4.48]; 58.00 {56.00–67.00}	64.81 [± 4.02]; 65.50 {57.00–70.00}	0.013
Mean intensity of spontaneous speech (dB), on-stimulation/on-medication	60.67 [± 5.77]; 58.00 {54.00–70.00}	67.87 [± 4.50]; 68.50 {55.00–74.00}	0.008
F0 SD of spontaneous speech (Hz) on-stimulation/off-medication	35.87 [± 14.03]; 31.66 {21.06–61.83}	34.71 [± 17.61]; 31.45 {14.98–80.24}	0.610
F0 SD of spontaneous speech (Hz) off-stimulation/off-medication	37.24 [± 23.43]; 30.79 {10.12–92.56}	25.98 [± 10.75]; 27.17 {7.84–44.20}	0.193
F0 SD of spontaneous speech (Hz) on-stimulation/on-medication	39.08 [± 13.33]; 31.92 {25.85–63.47}	29.26 [± 11.32]; 28.15 {13.87–46.79}	0.079
Maximum phonation time (MPT) (s) on-stimulation/off-medication	13.35 [± 6.28]; 11.00 {7.00–25.00}	16.38 [± 7.33]; 15.00 {6.20–32.00}	0.173
Maximum phonation time (MPT) (s) off-stimulation/off-medication	9.85 [± 4.34]; 9.00 {5.00–18.00}	13.67 [± 5.11]; 12.50 {8.60–27.00}	0.064
Maximum phonation time (MPT) (s) on-stimulation/on-medication	11.77 [± 2.90]; 12.00 {7.00–18.00}	14.84 [± 6.30]; 14.50 {7.00–31.00}	0.156
Mean intensity of sustained phonation (dB), on-stimulation/off-medication	63.00 [± 7.81]; 64.00 {52.00–76.00}	72.06 [± 8.38]; 71.50 {56.00–88.00}	0.020
Mean intensity of sustained phonation (dB), off-stimulation/off-medication	63.22 [± 6.44]; 63.00 {55.00–74.00}	69.72 [± 6.71]; 68.50 {60.00–85.00}	0.031
Mean intensity of sustained phonation (dB), on-stimulation/on-medication	65.55 [± 10.28]; 71.00 {46.00–75.00}	71.31 [± 7.58]; 70.50 {60.00–88.00}	0.364
Count rate (sill/s) on-stimulation/off-medication	4.49 [± 1.80]; 4.64 {2.22–7.28}	4.16 [± 1.22]; 4.00 {2.68–6.60}	0.798
Count rate (sill/s) off-stimulation/off-medication	4.15 [± 1.47]; 4.25 {1.46–6.37}	4.16 [± 0.99]; 4.44 {2.55–5.66}	0.977
Count rate (sill/s) on-stimulation/on-medication	5.25 [± 2.35]; 4.64 {2.32–9.44}	4.64 [± 1.14]; 4.44 {2.22–7.28}	0.691
Speech intelligibility (%), on-stimulation/off-medication	73.78 [± 17.46]; 78.00 {48.00–94.00}	98.37 [± 2.45]; 100.00 {92.00–100.00}	0.000
Speech intelligibility (%), off-stimulation/off-medication	66.78 [± 18.71]; 64.00 {48.00–94.00}	94.22 [± 6.52]; 96.00 {78.00–100.00}	0.000
Speech intelligibility (%), on-stimulation/on-medication	69.56 [± 22.53]; 80.00 {28.00–92.00}	93.12 [± 7.59]; 96.00 {76.00–100.00}	0.001
Perceptual severity of dysarthria (1–4) on-stimulation/off-medication	2.88 [± 0.78]; 3.00 {1.00–4.00}	3.68 [± 0.47]; 4.00 {3.00–4.00}	0.005
Perceptual severity of dysarthria (1–4) off-stimulation/off-medication	2.88 [± 0.78]; 3.00 {2.00–4.00}	3.67 [± 0.49]; 4.00 {3.00–4.00}	0.010
Perceptual severity of dysarthria (1–4) on-stimulation/on-medication	3.00 [± 0.86]; 3.00 {2.00–4.00}	3.43 [± 0.51]; 3.00 {3.00–4.00}	0.191
Trail making test part B	242.71 [± 146.45]; 232.00 {74.00–531.00}	116.80 [± 95.23]; 97.50 {27.00–310.00}	0.05

*PD* Parkinson disease, *LEDD* Levodopa Equivalent Daily Dose, *PIGD* dominant postural instability and gait disorder, *SD* standard deviation, *UPDRS* Unified Parkinson's Disease Rating Scale.

### Secondary outcomes

Regarding the comparison between the different speech variables in the conditions tested, a statistically significant reduction of the duration of sustained phonation was found in the off-stimulation/off-medication condition when compared with the on-stimulation/off-medication condition (p < 0.005) and with the preoperative on-medication condition (p < 0.005). In addition, the mean intensity of sustained phonation was significantly reduced in the off-stimulation/off-medication condition if compared with the preoperative off-medication condition (p < 0.005) highlighting the negative effects of disease progression on this acoustic parameter.

A direct correlation between the variation of speech intelligibility and tremor (p = 0.026) and PIGD subscores (p = 0.040) by comparing the off-stimulation/off-medication with the on-stimulation/off-medication one was also found, highlighting that the rebound of tremor and axial symptoms can worsen speech in the acute testing situation. On the contrary, no correlations were found between the variation of speech intelligibility and the changes in the different motor scores and subscores when comparing the on-stimulation/off-medication and preoperative off-medication condition. Furthermore, a negative correlation was also found in the on-stimulation/off-medication condition between speech intelligibility and akinesia subscore (p = 0.025) meaning that patients with a more severely decreased movements amplitude and speed have less intelligibility. The negative correlation between speech intelligibility and akinesia subscore was also confirmed in the on-stimulation/on-medication condition (p = 0.013).

## Discussion

Our results add to further observations regarding the long-term effects of bilateral STN-DBS on speech in PD patients^7,12^. Globally, the studied cohort showed a long-term maintenance of speech intelligibility after surgery, highlighting some possible beneficial long-term effects of STN-DBS when comparing off stimulation and on stimulation conditions. This is consistent with previous studies that showed no worsening of speech following STN-DBS when assessed either with the UPDRS speech item^15^ or dedicated perceptual assessments^16^, which was reduced with the introduction of L-DOPA^17^, in line with our findings. Nevertheless, when using a proper perceptual assessment of speech intelligibility, it is commonly accepted that a significant worsening of speech intelligibility should be expected one year after surgery, when comparing postoperative on-stimulation/off-medication condition with the preoperative off-medication condition^9,18^. This has also been reported in the long-term by Aviles-Olmos et al.^12^, who reported a significant worsening of speech intelligibility at five (− 43.7%) and eight years (− 21.4%) in the off-medication conditions if compared with preoperative values. The different results obtained in our cohort may be due to the different evaluation of speech intelligibility (i.e., sentence task of the Assessment of Intelligibility for Dysarthric Speech and single word intelligibility). However, by looking at the individual cases of our cohort in detail, two distinct subgroups could be identified based on the long-term changes of speech intelligibility, with different clinical characteristics. This is in line with similar approaches that tried to disentangle different possible speech outcomes following STN-DBS^18,19^. Patients in the “worsened” subgroup showed a worse preoperative and postoperative motor disease severity together with a worse cognitive function particularly regarding executive-frontal domain. These results are in line with a recent 2-year follow-up study that underlined the potential predictor role of preoperative cognitive function for speech deterioration after STN-DBS^19^. The trend toward the significance of PIGD subscores between the two “worsened” and “stable” subgroups suggests a possible link between speech deterioration and axial features, confirming the correlation between hypokinetic dysarthria and PD axial symptoms^8^. A similar tendency towards significance was also found for the over-distribution of GBA1-PD patients in the “worsened” group. GBA1-PD patients complain about higher axial and cognitive burden when compared with wild-type PD patients^20^ leading to the assumption that GBA1-PD patients may be at higher risk of developing post-operatively not only gait and cognitive worsening but also speech deterioration. Interestingly, disease duration, sex, duration of follow-up and the total electrical energy delivered by STN-DBS^21^ did not significantly differ between the two groups. This result is in line with a previous study that did not find any association between speech intelligibility deterioration and disease duration^7^ even if another study by Tripoliti et al.^9^ found that disease duration was a predictive factor for speech outcome. In addition, concerning preoperative speech variables we did not find differences between the two groups, while postoperatively, both speech intelligibility and mean intensity of spontaneous speech were significantly lower in the worsened group if compared with the stable one. With regards to the comparison between the different speech variables, a statistically significant reduction of the intensity of sustained phonation emerged in the off-stimulation/off-medication condition if compared with the on-stimulation/off-medication condition highlighting the possible positive effect of STN-DBS on voice intensity, as already confirmed in previous studies^7,18^. The improvement of speech intensity due to STN-DBS has been linked to its effects on hypokinesia and rigidity of language-related organs^18,22^ which represent one of the major pathophysiological bases of hypokinetic dysarthria, characterized by hypophonia, monotony, hypoarticulation of consonants and inappropriate silences^23^.

A further result that emerged in our sample was the significant reduction of the maximum phonation time (MPT) in the off-stimulation/off-medication condition when compared to the on-stimulation/off-medication condition. This result shows how stimulation, even 5 years after surgery, can positively influence MPT that is significantly reduced following the device’s shutdown. This finding is in line with previous studies that reported a beneficial effect of stimulation on MPT in the short-term after surgery^24^. Even in this case, the reduction of MPT can be traced back to the underlying laryngeal dysfunction and the reduced respiratory volume mainly related to the rigidity and hypo-bradykinesia of the laryngeal and diaphragmatic muscles^23^.

This study also allows for evaluation of the possible effects of dopaminergic therapy alone on speech parameters by comparing the preoperative off-medication and on-medication conditions. The results obtained did not show statistically significant differences between these two conditions, suggesting that dopaminergic therapy alone did not significantly improve or maintain speech postoperatively. These findings were consistent with previous studies that showed little or no effects of levodopa alone on acoustic speech parameters^8,25–27^. In particular, no significant changes following levodopa intake were found in phonatory, articulatory or prosodic parameters, as previously reported^27^ confirming the possible involvement of non-dopaminergic pathways in the pathophysiology of hypokinetic dysarthria in PD^8^.

We also found a direct correlation between the variation of speech intelligibility and tremor and PIGD subscores highlighting that the rebound of tremor and axial symptoms may worsen speech in the acute testing situation when stimulation is turned off. In addition, speech intelligibility also correlated with the UPDRS akinesia subscore in two out of the three postoperative conditions tested, highlighting a possible link between the decrease in limb movement speed and amplitude and speech. This is in line with other studies that have reported a correlation between speech variables and limb bradykinesia^28,29^. Our study has several limitations, including a lack of definition for the position of the electrodes, the small sample size, the lack of a control group and the lack of the assessment of speech intelligibility also at the sentence level. Nevertheless, our findings highlight the possibility of positive effects of STN-DBS on speech intelligibility after surgery in the long-term. Interestingly, it also gives a better understanding of PD characteristics associated with long-term speech worsening after STN-DBS. This information may allow clinicians to improve candidates’ selection for DBS and refine prognostic accuracy (e.g., GBA1 genetic status influence on speech) and that, if necessary, early speech interventions should be used after surgery in PD patients treated with STN-DBS at a higher risk of speech deterioration.

## Methods

### Participants

Patients treated with bilateral STN-DBS from 2012 to 2017 at the Neurological Unit of the OCB University Hospital were included. All patients fulfilled the diagnosis of PD according to the UK Brain Bank criteria^30^ and suffered from disabling motor complications. Data from non-native Italian speakers were excluded. This study was approved by the ethics committee of the Area Vasta Emilia Nord, Italy (protocol number: 2019/0,056,629), and written informed consent was obtained from participants. The study was performed in accordance with the Declaration of Helsinki.

### Clinical assessment

The clinical evaluation was performed in accordance with the CAPSIT-PD protocol^31^. Levodopa responsiveness was evaluated through an acute levodopa challenge. Hoehn and Yahr scale (H&Y) and the Unified Parkinson’s Disease Rating Scale (UPDRS)^32^ were applied to quantify disease severity both in the “off-medication” condition (obtained after a 12-h antiparkinsonian medication withdrawal) and “on-medication” condition (obtained after 60 min and the administration of a 30% higher dose of the usual levodopa morning intake)^31^. Different subscores were extrapolated from the UPDRS including tremor, postural instability/gait disorders (PIGD), akinesia and UPDRS item 18 (speech) subscores. PD motor phenotype (tremor dominant [TD], indeterminate and PIGD)^33^ was also extrapolated and patients were screened for the presence of mutations in the glucocerebrosidase-1 (GBA-1), leucine-rich repeat kinase-2, α-synuclein and parkin genes^34,35^. The total amount of dopaminergic medications was calculated as levodopa equivalent daily dose (LEDD) milligrams^36^. All patients underwent 3-Tesla brain-MRI to evaluate the presence/absence of white matter hyperintensities of vascular origin. All subjects were re-evaluated with a median five-years follow-up (range 3–7 years) after surgery. Neurological evaluation (superimposable with the preoperative one) and the speech assessment were carried out on the same day and in the following conditions: on-stimulation/off-medication (washout of at least 12-h of dopaminergic medications); off-stimulation/off-medication (stimulation was temporarily turned off for at least 1-h); on-stimulation/on-medication (stimulation was turned on and dopaminergic therapy was administered [early morning LEDD plus 30%]). Each patient underwent a complete neuropsychological assessment before surgery and at long-term evaluation, including phonemic fluency, spatial perception (localization of numbers), Raven’s progressive matrices, Stroop test and Trail making test part B.

### Speech evaluation

Speech evaluation was performed both preoperatively during the acute levodopa challenge and at postoperative assessment. Each evaluation was carried out in a silent environment with conversation voice intensity and was recorded with a digital voice recorder (model SONY ICDPX240) kept 20 cm from the patient’s lips^25^. The perceptual-acoustic analysis was performed using Praat software^37^ and blinded to the patient’s condition. The following tasks were included: word intelligibility (calculated as the percentage of words correctly transcribed by the examiner among a set of 25 recorded words)^25^; oral diadochokinesis task in which participants produced the syllables /pa/, /ta/, /ka/ and the pseudoword /pataka/, as fast as they could with habitual pitch and loudness (irregular rhythm [presence of absence], uncontrolled acceleration [presence of absence]); sustained production of the phoneme /a/ for as long as possible, performed three times (duration [sec], intensity [dB]); counting from 1 to 20 (speech rate [syllables/second]). Single words intelligibility was selected due to its advantage of eliminating a number of other variables that can affect intelligibility, such as sentence level syntactic and prosodic variables. Furthermore, the use of single words to assess intelligibility is a much less difficult task for dysarthric participants than sentence level productions. As such, if an intelligibility impairment is noted at the single word level, intelligibility deficits are more than likely at higher/more complicated levels of speech productions, such as the sentence level^38^. A calibration tone (80 SPL dB, 1 kHz) was included at the beginning of each recording to serve as a reference in the determination of speaking amplitude. According to recent guidelines^39^, these parameters have been selected because they represent acoustic characteristics previously reported as altered in hypokinetic dysarthria^23,27^. The presence and severity of hypokinetic dysarthria were perceptually determined by two speech language pathologists, both Italian native speakers. Speakers’ severity of dysarthria was categorized on a coarse scale ranging from none, mild, moderate to severe (1: severe, 2: moderate; 3: mild: 4: none)^40,41^.

### Statistical analysis

Descriptive statistics were used for describing demographic and clinical data. The primary objective of the study was to evaluate the long-term effects of bilateral STN-DBS on speech intelligibility in advanced PD patients. As primary outcomes, we selected the change of speech intelligibility between postoperative on-stimulation/off-medication condition and preoperative off-medication condition; postoperative on-stimulation/off-medication and off-stimulation/off-medication condition. Positive changes represented improvement of speech intelligibility while negative changes represented speech worsening. The presence of significant differences in speech intelligibility in the different conditions tested was calculated using the Friedman test with subsequent post-hoc Wilcoxon signed rank test with Bonferroni correction for multiple comparisons because of the use of multiple tests (statistical adjusted significance was set at p-value < 0.005). Based on the presence/absence of long-term postoperative worsening of speech intelligibility, patients were divided into two groups (“stable/imp” [absent or positive variation] and “worsened” [negative variation]) that were compared to find significant differences in demographic, clinical and speech variables. With regards to continuous and ordinal variables, the Mann–Whitney test was used, while for categorical variables the chi-square independence test was applied (statistical significance was set at p-value < 0.05). Secondary outcomes included: the changes of the other speech variables in the different conditions tested; the correlation between the variation of speech intelligibility and the variation in the different motor scores and subscores in the on-stimulation/off-medication condition compared with the off-stimulation/off-medication condition and in the on-stimulation/off-medication compared with preoperative off-medication condition; the correlation between the different motor scores and subscores and speech intelligibility in the postoperative conditions tested. Correlation analyses were performed by using the Spearman Correlation analysis. Statistical analysis was performed using the IBM SPSS Statistics for Windows version 20.0 (IBM, Armonk, NY, USA).

## Data Availability

Anonymized data of this study may be available from the corresponding author upon request from any qualified researcher, following the EU General Data Protection Regulation.
