# Author Correction: Long-term effects of subthalamic nucleus deep brain stimulation on speech in Parkinson’s disease

**DOI:** 10.1038/s41598-023-39958-x

**Published:** 2023-08-17

**Authors:** Annalisa Gessani, Francesco Cavallieri, Valentina Fioravanti, Isabella Campanini, Andrea Merlo, Giulia Di Rauso, Benedetta Damiano, Sara Scaltriti, Elisa Bardi, Maria Giulia Corni, Francesca Antonelli, Francesca Cavalleri, Maria Angela Molinari, Sara Contardi, Elisa Menozzi, Alessandro Fraternali, Annibale Versari, Giuseppe Biagini, Valérie Fraix, Serge Pinto, Elena Moro, Carla Budriesi, Franco Valzania

**Affiliations:** 1 Neurology Unit, Department of Neuroscience, S. Agostino Estense Hospital, Azienda Ospedaliero-Universitaria di Modena, Modena, Italy; 2 Neurology Unit, Neuromotor & Rehabilitation Department, Azienda USL-IRCCS di Reggio Emilia, Viale Risorgimento 80, 42123 Reggio Emilia, Italy; 3 Clinical and Experimental Medicine PhD Program, University of Modena and Reggio Emilia, Modena, Italy; 4 LAM – Motion Analysis Laboratory, Neuromotor and Rehabilitation Department, San Sebastiano Hospital, Azienda USL-IRCCS di Reggio Emilia, Correggio (Reggio Emilia), Italy; 5 Division of Neuroradiology, Department of Neuroscience, Nuovo Ospedale Civile S. Agostino Estense, Modena, Italy; 6 Department of Neurology and Stroke Center, IRCCS Istituto Delle Scienze Neurologiche di Bologna, Maggiore Hospital, Bologna, Italy; 7 Department of Clinical and Movement Neurosciences, UCL Queen Square Institute of Neurology, London, UK; 8 Nuclear Medicine Unit, Azienda Unità Sanitaria Locale-IRCCS di Reggio Emilia, Reggio Emilia, Italy; 9 Department of Biomedical, Metabolic and Neural Sciences, University of Modena and Reggio Emilia, Modena, Italy; 10 Grenoble Alpes University, Division of Neurology, Centre Hospitalier Universitaire de Grenoble, Grenoble Institute of Neuroscience, Grenoble, France; 11 Aix-Marseille Univ, CRNS, LPL, Aix-en-Provence, France

Correction to: *Scientific Reports* https://doi.org/10.1038/s41598-023-38555-2, published online 15 July 2023

The Acknowledgements section in the original version of this Article was incomplete.

“Thanks are due to Mr. Jovian Salak for the English revision of the manuscript.”

now reads:

“This study was partially supported by Italian Ministry of Health – Ricerca Corrente Annual Program 2023. Thanks are also due to Mr. Jovian Salak for the English revision of the manuscript."

The original Article has been corrected.

